# The Effect of Larval Exposure to Plastic Pollution on the Gut Microbiota of the Major Malaria Vector *Anopheles arabiensis* Patton (Diptera: Culicidae)

**DOI:** 10.1111/1758-2229.70169

**Published:** 2025-08-03

**Authors:** Shristi Misser, Chia‐Yu Chen, Arshad Ismail, Shüné V. Oliver

**Affiliations:** ^1^ Faculty of Health Sciences Wits Research Institute for Malaria, University of the Witwatersrand Johannesburg South Africa; ^2^ Division of the National Health Laboratory Service, Centre for Emerging Zoonotic and Parasitic Diseases National Institute for Communicable Diseases Johannesburg South Africa; ^3^ Sequencing Core Facility, Division of the National Health Laboratory Service National Institute for Communicable Diseases Johannesburg South Africa; ^4^ Department of Biochemistry and Microbiology, Faculty of Science, Engineering and Agriculture University of Venda Thohoyandou South Africa; ^5^ Institute for Water and Wastewater Technology, Durban University of Technology Durban South Africa

**Keywords:** bioinformatics, environmental signal/stress responses, microbiome, pollution microbiology

## Abstract

Plastic pollution is prevalent in water bodies. However, most studies on plastic pollution focus on marine environments, with limited knowledge about its impact on freshwater ecosystems. This paucity of information extends to the effect on aquatic insects, with little reported data on the effect of plastic on malaria vectors. This is concerning as microplastics are reported to perturb the gut microbiota of culicine mosquitoes. This study examines how larval exposure to degraded plastic, plastic additives (phthalic acid, Bisphenol‐A) and latex beads affects the gut microbiota of adult *Anopheles arabiensis*, with a comparison of the insecticide‐unselected (SENN) and insecticide‐selected (SENN‐DDT) strains. The larval exposure had a minimal effect on alpha‐diversity, but each plastic stressor altered beta‐diversity in a non‐strain–specific manner. Plastic‐treated SENN showed an increase in unique bacterial genera. In contrast, untreated SENN‐DDT displayed the highest abundance of unique genera, suggesting gut bacteria may play a role in mitigating the effect of plastic exposure in unselected strains. Additionally, larval plastic exposure increased bacteria associated with plastic degradation and pesticide metabolism. Although there was no significant change in *Plasmodium*‐protective bacterial genera, inflammation‐associated bacterial genera increased in both strains after treatment, suggesting potential immune modulation.

## Graphical abstract

Two laboratory strains of *Anopheles arabiensis* exposed to four plastic treatments resulted in minimal effects on alpha diversity and a non‐strain–specific alteration of beta diversity. Although no significant change to *Plasmodium‐*protective bacteria was observed, an increase in bacteria associated with plastic degradation, pesticide metabolism and inflammation was present after plastic treatment in both strains.

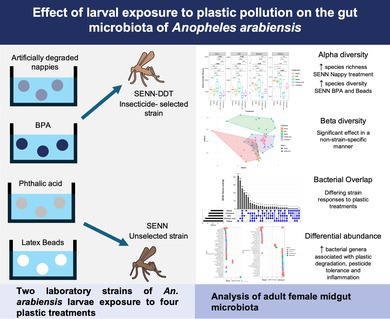

## 1 Introduction

Plastics are polymers that can be easily shaped and moulded. Plastics were initially developed from natural materials like latex from trees and natural rubber. Since the invention of the first synthetic plastic, Bakelite, in 1907, synthetic polymers have been used as the primary forms of plastic. Plastics were considered safe, clean and inexpensive materials which led to large‐scale use of these products (Meikle 1995).

A 2017 review estimated the total amount of plastic produced to date was 8.3 billion tons. Furthermore, it is estimated that 60% of plastic ever produced has been discarded and remains in landfills (Geyer et al. 2017). As such, waste plastic is subject to degradation, usually by hydrolysis, photodegradation, thermooxidative degradation or biodegradation by microbes. The biodegradation of plastic waste by oxidative conditions, UV radiation, physical stress and microbes results in the breakdown of these polymers into mesoplastics (between 5 mm and 2.5 cm), microplastics (< 5 mm) and nanoplastics (< 1 μm) (Andrady 2011). These breakdown products, however, are not the only source of microplastics. Primary microplastics arise from plastics that are purposefully manufactured to be small‐sized to be added to products such as scrubbing agents. As such, domestic waste is a major source of primary microplastic pollution (Boucher and Friot 2017).

The most common plastic pollutants come from packing materials. These include polyethylene (38% of plastic production), polypropylene (24%), polystyrene (6%), polyethylene terephthalate and polyvinyl chloride (19%). Nylon and cellulose acetate also contribute substantially to plastic pollution (Andrady 2011). However, it is not only these polymers themselves that contribute to the pollution associated with plastic production. Additives such as plasticisers contribute to the pollution associated with plastics as well, often through the breakdown of the plastics. These include Bisphenol‐A (BPA) and phthalates (Nayanathara Thathsarani Pilapitiya and Ratnayake 2024).

Plastic pollution, whether microplastics, plastic components or plastic additives, has a range of health and environmental effects. There has been a major focus on the effect of marine plastic pollution (Napper and Thompson 2023; Nayanathara Thathsarani Pilapitiya and Ratnayake 2024). However, despite the ubiquity of plastic pollution, there is far less understanding of the effect of this type of pollution on freshwater sources, particularly on freshwater invertebrates. Most research on freshwater invertebrates has been performed on planktonic and benthic organisms, usually model organisms such as *Daphnia* (Jones et al. 2024). Furthermore, even recent reviews of literature on the effects of plastic pollution on freshwater invertebrates did not have a large focus on insects (Pirillo and Baranzini 2022). However, there have been reports of the effect of microplastics on the life history of mosquitoes, due to their importance as disease vectors.

The probability of exposure to plastic pollution varies depending on the distribution of mosquitoes. Mosquitoes associated with urban areas such as *Culex*, *Aedes* and *Anopheles stephensi* are more likely to be exposed to plastic pollution due to their association with human activity. Most *Anopheles* species would be exposed to pollutants through agricultural sources and therefore will be exposed to plastic at lower rates (Jones et al. 2024). It is therefore likely that there is less research on the effects of plastic exposure on *Anopheles*. The effects of microplastic exposure on mosquitoes are largely dependent on the size of the plastic particle, concentration and mosquito species. Variable effects have been seen on larval development (neutral to negative), and ontogenic transfer of microplastics is not guaranteed. This was observed by a comparison of the effect of plastic in *Aedes aegypti*, *Aedes albopictus*, *Culex pipiens*, *Culex quinquefasciatus* and *Culex tarsalis* (Jones et al. 2024). Where ontogenic transfer does occur, mosquitoes can transfer the microplastics through bites (J. Li et al. 2024). If microplastics are present in adults, mosquitoes can be potential mechanical vectors of these products and could potentially transfer them to new environments or trophic levels (Al‐Jaibachi et al. 2018).

In addition to the variability in the effects of microplastics on mosquito life history, there are two clear effects that exposure to microplastics can have on the mosquito. Microplastics can serve as sinks for persistent organic pollutants (Steinman et al. 2020; Napper and Thompson 2023). This can perturb the metabolome (Wang et al. 2022) and has been reported to reduce the efficacy of insecticides (J. Li et al. 2024). As some plastic additives are known endocrine disruptors, the chemical disruption is unsurprising (Napper and Thompson 2023).

Furthermore, there have been recent reports of microplastics affecting the mosquito microbiome (Edwards et al. 2023; J. Li et al. 2024). Factors that affect the mosquito microbiome are important due to their critical role in the organism's life history. The mosquito gut microbiome is critical for mosquito development (Coon et al. 2014, 2017), egg production (Coon et al. 2016), digestion and immunity (Gao et al. 2020). As such, larval exposure to plastic could have large‐scale effects on mosquito life history through the modulation of the microbiome. These large‐scale effects may lead to increased longevity and improved survivorship of the mosquito populations, which hold considerable epidemiological significance, particularly in terms of disease transmission.

Furthermore, the majority of research primarily focuses on the effect of microplastics on mosquito populations. However, it is essential to acknowledge that plastic pollution represents a multifaceted challenge, incorporating a range of physical and chemical properties associated with various types of plastics. The physical characteristics under consideration include size, shape and concentration of microplastics (Campanale et al. 2020). In addition, the chemical plastic additives added to plastics may exert significant effects as well. To attain a more comprehensive understanding of the implications of plastic pollution, it is imperative to extend investigations to encompass not only microplastics but also the associated chemical plastic additives. Consequently, the concept of plastic exposure must be broadly defined to include both microplastics and chemical plastic additives.

Although there have been recent reports of microplastic effects on mosquitoes, to the best of our knowledge, there have been no reports on the effects on African malaria vectors. This is a knowledge gap as anthropophilic members of the *Anopheles gambiae* complex are adapting to breeding in polluted waters which facilitates their spread to new habitats (Antonio‐Nkondjio et al. 2011). This adaptation is particularly true for *Anopheles arabiensis*, which is increasingly adapting to urban environments (Azrag and Mohammed 2018; Bimbilé Somda et al. 2018; Fournet et al. 2022). For *An. arabiensis*, this is problematic because this vector has extremely adaptable behaviour and is highly flexible in feeding habits (Sinka et al. 2010). Moreover, due to *An. arabiensis* predominantly biting outdoors, this species is difficult to control by conventional vector control activities, which typically focus on indoor biting mosquitoes (Kitau et al. 2012). As such, it is critical to understand the effect of plastic exposure on *An. arabiensis*.

## 2 Experimental Procedure

### 2.1 Strains Used in This Study

Two laboratory strains of *An. arabiensis* were used in this study. The SENN strain was colonised in 1980 from a strain originating from Gezira in Sudan (Hemingway 1983). SENN had low‐level deltamethrin resistance (Oliver and Brooke 2017), but at present, the strain is primarily insecticide‐susceptible. This strain, therefore, represents an insecticide‐unselected strain. From SENN, the SENN‐DDT strain was selected by continuous exposure to 4% DDT since 1995, which continues to the present day. SENN‐DDT displays resistance to DDT, permethrin, deltamethrin, λ‐cyhalothrin and malathion (Oliver and Brooke 2017). Resistance in SENN‐DDT is mediated by fixation of the L1014F mutation in the para‐sodium channel gene as well as enhanced metabolic detoxification enzymes (Oliver and Brooke 2013). Both strains were reared according to the methods described in Hunt et al. (2005). In brief, mosquitoes were reared at 25°C (±2°C) and 75% humidity (±5%). Mosquitoes were exposed to a 12:12 h light:dark photoperiod with a 30‐min dawn/dusk cycle. Larvae were fed a diet of Beano dog biscuits and brewer's yeast (3:1) ratio, and adults were allowed ad libitum access to 10% sucrose.

### 2.2 Larval Pollutant Exposure

A range of plastic pollutants was used in this study. Fluorescent latex beads (2 μm, Sigma Aldrich, catalogue number L0280) were used as a proxy for the size of microplastics. A volume of 7 μL of this commercial solution was used per litre of water. Disposable nappies (Huggies Extra Care Disposable Nappies, Kimberly Clarke, serial number 5029053548647) were used as complex plastic pollutants. This product consisted of wood pulp, sodium polyacrylate, polypropylene, polyethylene, adhesives, polyester, polyurethane elastics and polyolefin elastic. In order to produce a degraded product, a single nappy was placed in 2000 mL of reverse osmosis water and allowed to degrade for 3 months under the insectary conditions described earlier. The degraded sample was further split into two and diluted with an additional 1000 mL to produce the water that constituted the nappy sample.

The diphenylmethane derivative, BPA (Sigma Aldrich, catalogue number 80‐05‐7) is used in the production of polycarbonate plastics and would therefore be a common plastic pollutant and serves as a representative of plastic breakdown products. A final concentration of 12.5 μM was used per 1000 mL of reverse osmosis water.

Phthalic acid dimethyl ester (1,2‐benzenedicarboxylic acid) (Sigma‐Aldrich, catalogue number 131‐11‐3) is used as a plasticiser and is a common pollutant associated with the production of plastic and does leach into the environment. Although not a plastic breakdown product, it does serve as a pollutant associated with plastics. A final concentration of 59.5 μM was used per 1000 mL of reverse osmosis water.

In order to generate samples for sequencing, 100 first instar larvae (< 24 h after hatching) of each strain were reared in 1000 mL of each of the described plastic pollutants. Samples reared in untreated reverse osmosis water served as an untreated control. Larvae were fed a standardised amount of food, which was 1 mg/larvae/day until third instar larvae, three times daily (Oliver and Brooke 2013). The adults were collected upon emergence. The adults were allowed *ad libitum* access to 10% sucrose until the age of 3 days, when females were then collected for processing.

### 2.3 Sample Processing and DNA Extraction

Three‐day‐old non‐blood–fed females were cold‐killed and the external surface ethanol‐sterilised. The midguts were dissected under sterile conditions. Three midguts were pooled per sample and five samples were used per treatment. The samples were then subjected to DNA extraction using a Qiagen DNeasy blood and tissue kit (Qiagen, catalogue number 69506) according to the manufacturer's directions. The samples were not physically homogenised, but an overnight incubation was performed to allow full tissue dissolution. A commercial microbial DNA extraction control was included as an extraction positive control (ZymoBiomics, catalogue number D6300). A buffer‐only negative control was also included.

### 2.4 Next‐Generation Sequencing and Bioinformatics

Total genomic DNA was used as a template to amplify the V3–V4 hypervariable region of the bacterial 16S rRNA gene. The primer sequences were as follows: forward primer 5′ GTC TCG TGG GCT CGG AGA TGT GTA TAA GAG ACA GGA CTA CHV GGG TAT CTA ATC C 3′ and reverse primer 5′ TCG TCG GCA GCG TCA GAT GTG TAT AAG AGA CAG CCT ACG GGN GGC WGC AG 3′ (4 nmol Ultramer DNA Oligo 55 bases, Integrated DNA Technologies, Coralville, IA, USA). A positive control consisting of a commercial Microbial Community DNA Standard (ZymoBIOMICS, D6300) and two negative controls (a non‐template and an extraction buffer‐only) were also amplified by PCR. Samples were sequenced at the Core Sequencing Unit at the National Institute for Communicable Diseases (NICD), Johannesburg, South Africa, as described in Chen et al. (2024).

Bioinformatic analysis was performed as per (Chen et al. 2024). In brief, the Nextera adapter sequences were removed by trimGalore (v0.6.5‐1) and MultiQC (v1.6) was used for quality control. Clean reads were pre‐processed using the DADA2 package (v1.24.0) (Callahan et al. 2016), and all downstream analysis was performed in R studio (v4.2.1). The obtained amplicon sequence variants (ASVs) were assigned taxonomy and the ASV abundance estimates were determined using the SILVA reference database (v138; https://zenodo.org/record/4587955#.Y9JGXnZBxPY, accessed 10 February 2024). The phyloseq package (v1.40.0) (McMurdie and Holmes 2013) was used to create phyloseq data objectives from the DADA2 outputs for further analysis. Alpha diversity was analysed using the Chao1 and ACE indices to assess species richness, while species diversity was assessed by the Shannon and Simpson diversity indices. The alpha diversity between the treatments was compared using the Wilcoxon rank‐sum test. Ordination plots to assess beta diversity were constructed using the Nonmetric Multidimensional Scaling (NMDS) method. The data clustering for the NMDS plot was statistically assessed using a PERMANOVA (permutation test with pseudo‐F ratios). Relative abundance for the top five genera was displayed in bar plots. UpSet plots to display shared microbial communities were generated using Venn Diagram (v1.7.3) and UpsetR (v1.4.0) (Conway et al. 2017). Differential abundance analysis between sample groups was performed using DESeq2 (v1.24.0) (Love et al. 2014).

## 3 Results

### 3.1 Alpha Diversity

**FIGURE 1:**
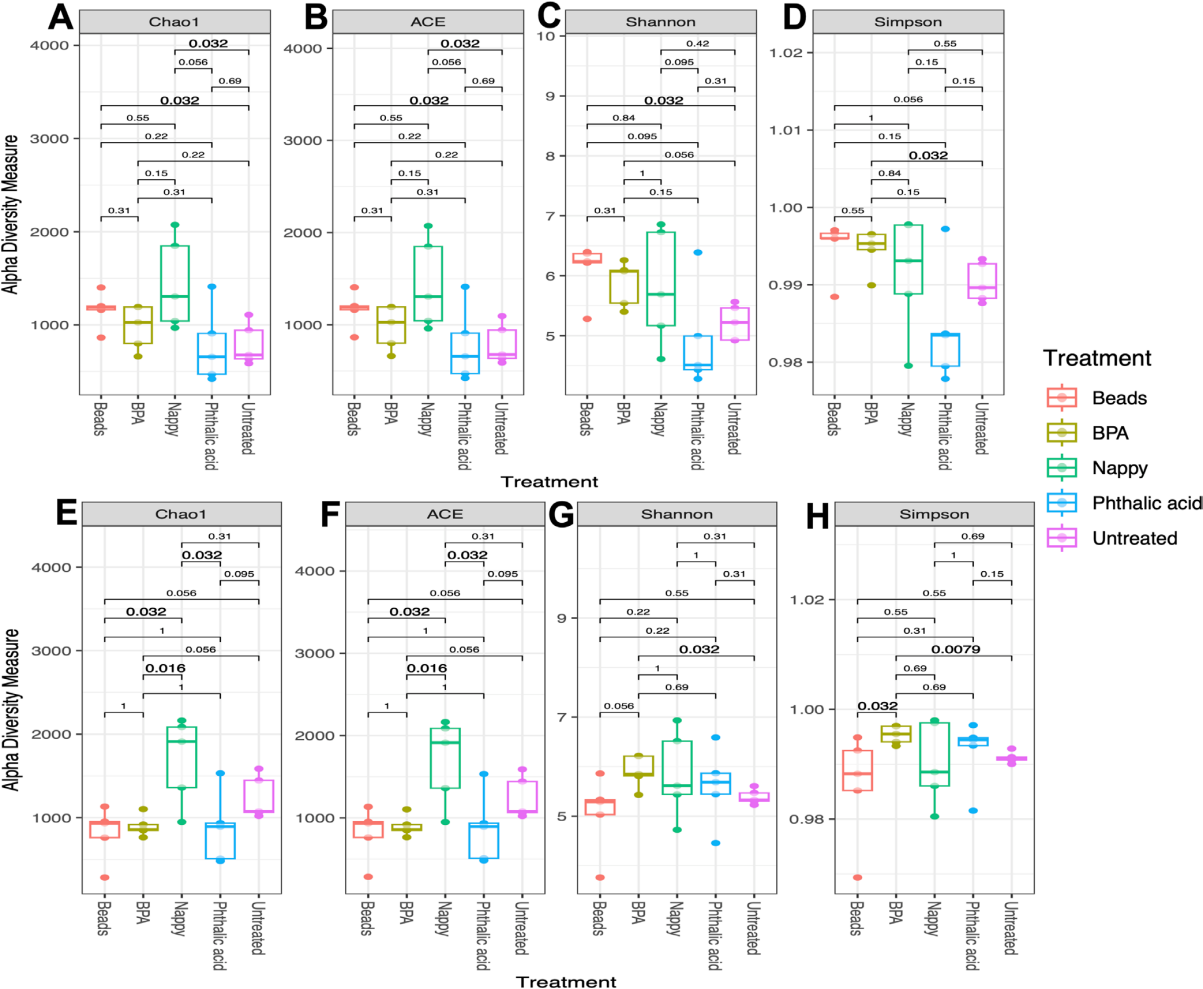
Alpha diversity in *Anopheles arabiensis* adults reared in plastic‐treated water. (A) Species richness measured by Chao1 index in SENN. (B) Species richness measured by ACE index in SENN. (C) Species diversity measured by Shannon index in SENN. (D) Species diversity measured by Simpson index in SENN. (E) Species richness measured by Chao1 index in SENN‐DDT. (F) Species richness measured by ACE index in SENN‐DDT. (G) Species diversity measured by Shannon index in SENN‐DDT. (H) Species diversity measured by Simpson index in SENN‐DDT. The *p*‐values were determined by the Wilcoxon rank‐sum test. Significant *p*‐values (< 0.05) are shown in larger text.

Rarefaction analysis highlighted a suitable sequencing depth. This is depicted in Supplementary Figure S1. The Chao1 and ACE indices represent bacterial species richness (the total number of species in a given sample). Both of these indices are sensitive to rare OTUs. For the SENN strain, the nappy and bead treatment had a significantly higher species richness than the untreated samples. This was true for the Chao1 index (*p* = 0.032) (Figure 1A) and ACE (*p* = 0.032) (Figure 1B). Species diversity was assessed using the Shannon and Simpson indices. In SENN, only the bead treatment had a higher Shannon diversity index than the untreated SENN (*p* = 0.032), with no further significant differences between the treatments (Figure 1C). For the Simpson index, the BPA treatment was significantly more diverse than the untreated sample (*p* = 0.032) (Figure 1D). This was the only difference in the treatment.

For the SENN‐DDT samples, there was a slight difference in the bacterial richness indices compared to SENN. The differences were between the treatment groups rather than compared to the untreated control. For the Chao1 index, the nappy treatment had a significantly greater species richness than the phthalic acid treatment (*p* = 0.032), BPA treatment (*p* = 0.016) as well as the bead treatment (*p* = 0.032) (Figure 1E). Similarly, for the ACE index, the nappy treatment was significantly higher than phthalic acid (*p* = 0.032), beads (*p* = 0.032) and BPA (*p* = 0.016) (Figure 1F). In terms of species diversity, the only significant difference in the Shannon index was an increased diversity in the BPA treatment compared to the untreated samples (*p* = 0.032) (Figure 1G). In the Simpson index, again, the BPA treatment had a significantly higher diversity than the untreated sample (*p* = 0.008) (Figure 1H). BPA treatment was also significantly more diverse than the bead treatment in terms of the Simpson index (*p* = 0.032) (Figure 1H).

### 3.2 Beta Diversity

**FIGURE 2:**
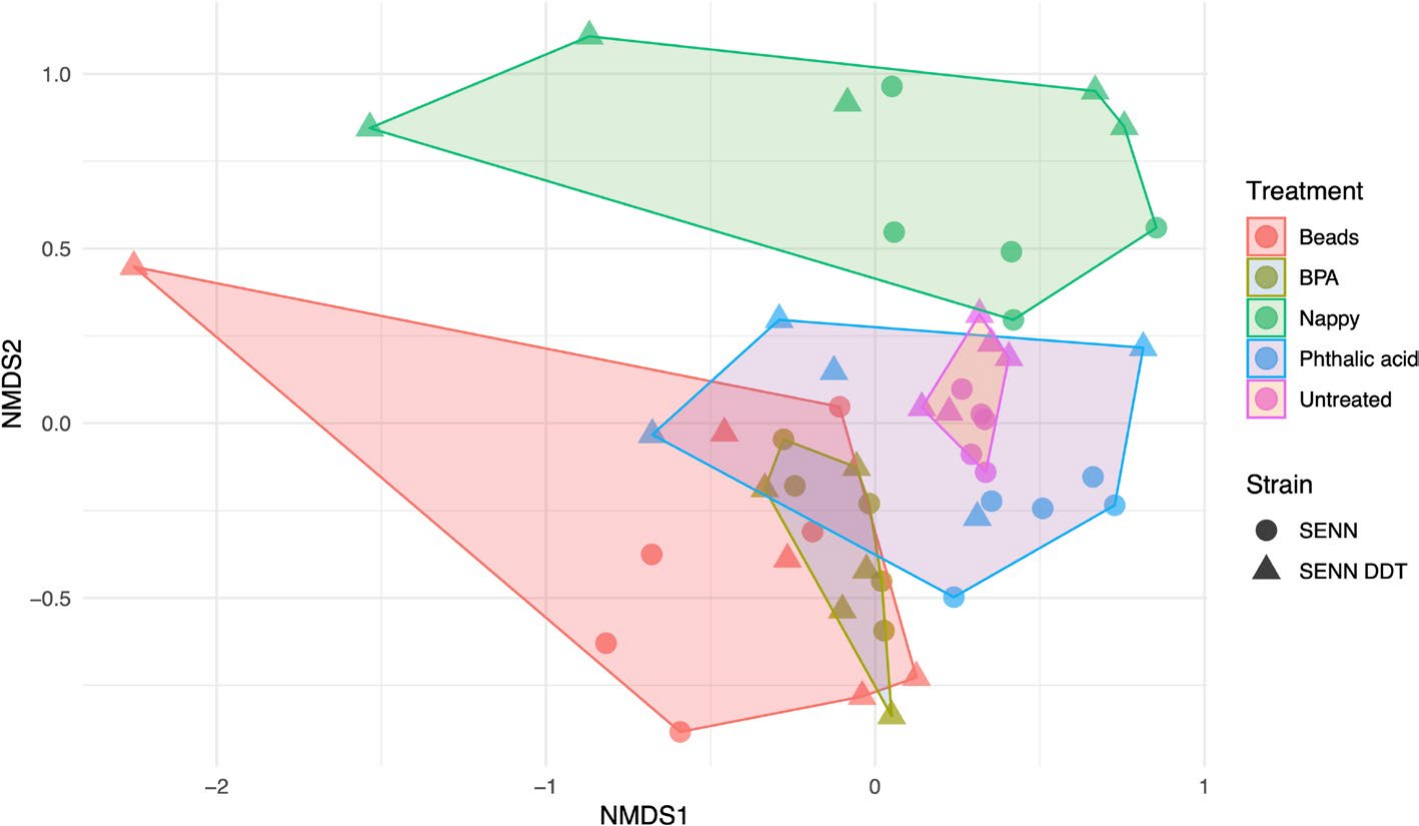
Beta diversity in *Anopheles arabiensis* adults reared in plastic‐treated water. Nonmetric Multidimensional Scaling (NMDS) ordination of diversity in SENN and SENN‐DDT adults bred in water treated with plastics. The NMDS plot was based on the Bray–Curtis distance measurements. The stress value was 0.19. A PERMANOVA indicated *p* < 0.001 for treatments, *p* = 0.05 for strain and *p* < 0.001 for treatment and strain. Circles represent the SENN strain and triangles represent SENN‐DDT.

Beta diversity, assessed here by NMDS analysis, was a measure of diversity between different *An. arabiensis* strains and plastic treatments (Figure 2). Larval exposure to plastic treatments had a significant effect on the beta diversity of the adults (PERMANOVA, *p* < 0.001, *R* ^2^ = 0.256). Although there was not a significant effect of strain (*p* = 0.050, *R* ^2^ = 0.023), there was a significant effect of interaction between strain and treatment (*p* < 0.001, *R* ^2^ = 0.104).

### 3.3 Relative Abundance

**FIGURE 3:**
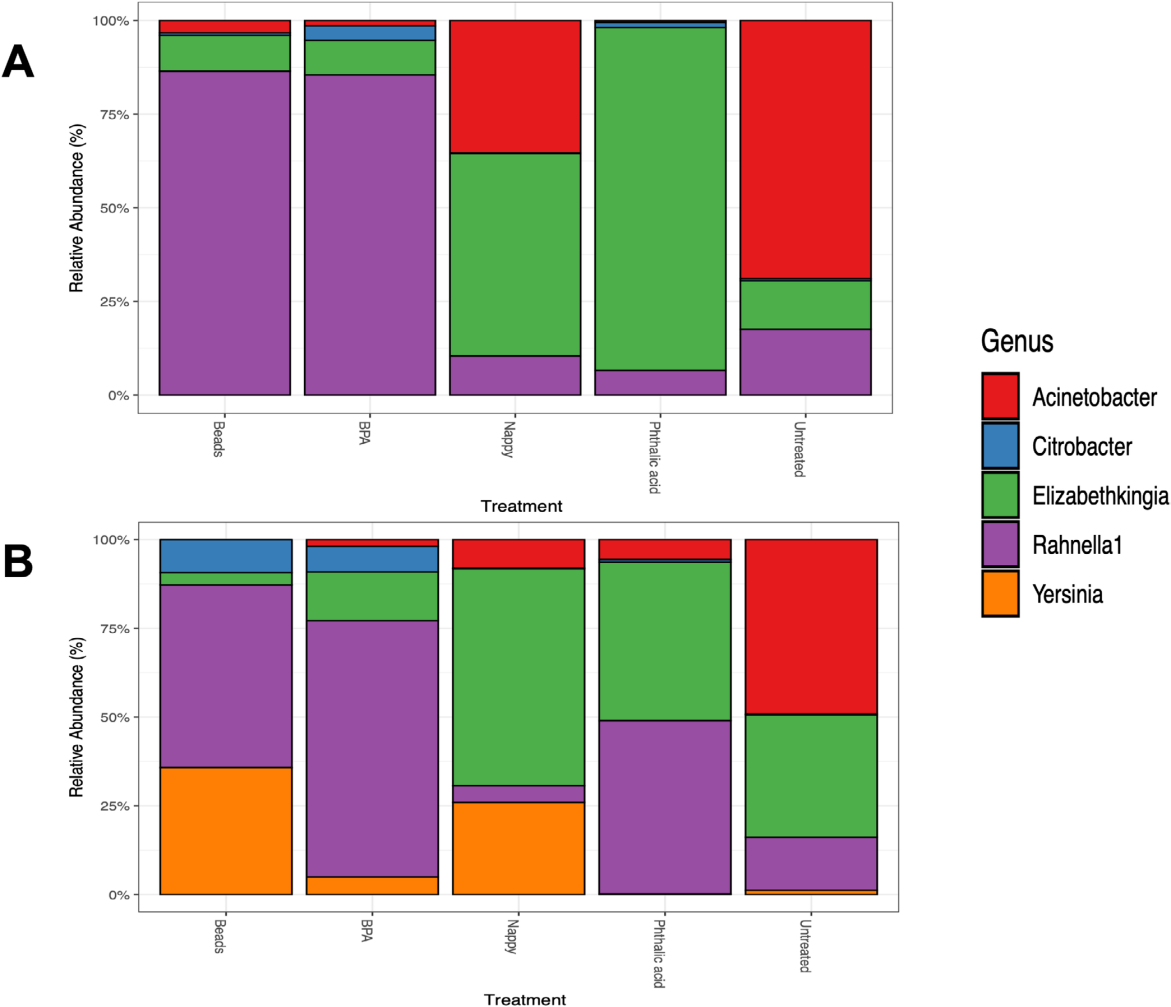
Relative abundance of the top five most abundant bacteria in *Anopheles arabiensis* adults reared in plastic‐treated water. (A) Relative abundance of SENN adults reared in water treated with beads, BPA, nappies, phthalic acid or untreated water. (B) Relative abundance of SENN‐DDT adults reared in water treated by beads, BPA, nappies, phthalic acid or untreated water.

The five most abundant bacterial genera in both strains were *Acinetobacter*, *Citrobacter*, *Elizabethkingia*, *Rahnella* and *Yersinia*. However, the proportions of the bacteria differ by treatment. In untreated SENN, *Acinetobacter*, *Rahnella* and *Elizabethkingia* dominated. In SENN treated with beads and BPA, *Rahnella* and *Elizabethkingia* were the dominant genera. *Elizabethkingia* dominated in nappy‐treated and phthalic acid–treated SENN, particularly in the latter. However, the next most dominant genera in the nappy‐treated SENN were *Acinetobacter* and *Rahnella* in the phthalic acid treatment (Figure 3A).

Although the patterns were similar in SENN‐DDT, the proportions differed. Notably, *Yersinia* was more present in SENN‐DDT than SENN. Untreated SENN‐DDT, like SENN, was dominated by *Acinetobacter*, *Rahnella* and *Elizabethkingia* but in different proportions. *Acinetobacter* and *Rahnella* were more evenly distributed in untreated SENN‐DDT than in SENN. Like in SENN, the beads‐ and BPA‐treated SENN‐DDT were more similar to each other than other treatments, where they were both dominated by *Rahnella*. However, the next most abundant genera in the bead‐treated SENN‐DDT was *Yersinia* while for BPA‐treated SENN‐DDT it was *Elizabethkingia*. Similar proportions of *Citrobacter* were present in these two treatments. *Elizabethkingia* dominated the nappy‐treated and phthalic acid–treated SENN‐DDTs, but the second most dominant genera in the nappy‐treated SENN‐DDT was *Yersinia*, while it was *Rahnella* in the phthalic acid–treated SENN‐DDT. *Acinetobacter* proportions were similar in the nappy‐treated and phthalic acid–treated SENN‐DDT. *Yersinia* was not present in the phthalic acid–treated SENN‐DDT (Figure 3B).

### 3.4 Overlapping Species

**FIGURE 4:**
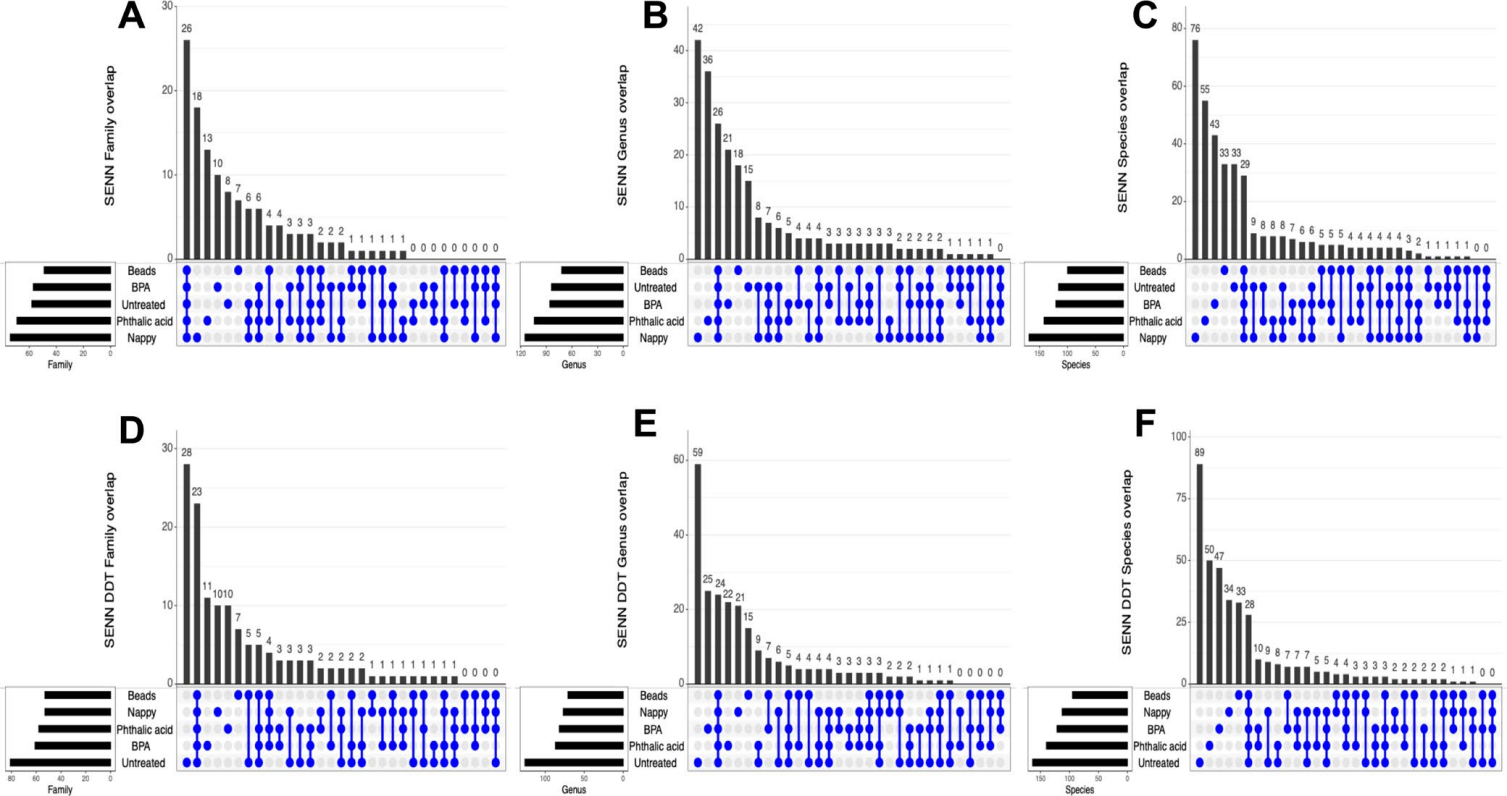
Upset plots displaying overlapping bacteria in *Anopheles arabiensis* adults reared in plastic‐treated water. (A) Overlapping families in SENN. (B) Overlapping genera in SENN. (C) Overlapping species in SENN. (D) Overlapping families in SENN‐DDT. (E) Overlapping genera in SENN‐DDT. (F) Overlapping species in SENN‐DDT. Individual dots indicate numbers of individual bacterial families, genera or species. Joined dots indicate the numbers of shared bacterial families, genera or species between the different plastic treatments.

An Upset Venn data analysis was performed to find the shared and unique bacterial families, genera and species for each strain after exposure to different plastic treatments. In SENN, there were 26 shared families between all treatments. The most unique genera were present in the nappy treatment (18), followed by phthalic acid (13), BPA (10), untreated (8) and beads (7) (Figure 4A). In terms of bacterial genera, nappies and phthalic acid treatments result in more unique bacterial species (42 and 36, respectively) compared to the 26 genera shared by all treatments. BPA‐treated SENN had 21 unique genera, beads‐treated had 18 and the untreated had 15 unique genera (Figure 4B). There were 29 shared bacterial species shared across all treatments against SENN. Nappy treatment of SENN resulted in 76 unique species, phthalic acid resulted in 55 unique species, BPA resulted in 43 unique species, while the untreated and beads‐treated SENN had 33 unique species, respectively (Figure 4C).

In the SENN‐DDT, the patterns were different. The untreated SENN‐DDT had the most unique bacterial families (28), while there were 23 shared families across all treatments. BPA‐treated SENN‐DDT had 11 unique families, while phthalic acid–treated and nappy‐treated SENN‐DDT both had 10 unique families. There were seven unique families in the beads‐treated SENN‐DDT (Figure 4D). In terms of bacterial genera, again the untreated SENN‐DDT had the most unique genera (59). There were 24 genera shared between the treatments. BPA‐treated SENN‐DDT had 25 unique genera, phthalic acid had 22 unique genera, nappy treatment had 21 unique genera and the beads had 15 unique genera (Figure 4E). There were fewer species shared between all the treatments (28) than the unique species in each treated SENN‐DDT. There were 89 unique species in the untreated SENN‐DDT group, 50 in the phthalic acid, 47 in the BPA treatment, 34 in the nappy and 33 in the beads (Figure 4F).

### 3.5 Differential Abundance

**FIGURE 5:**
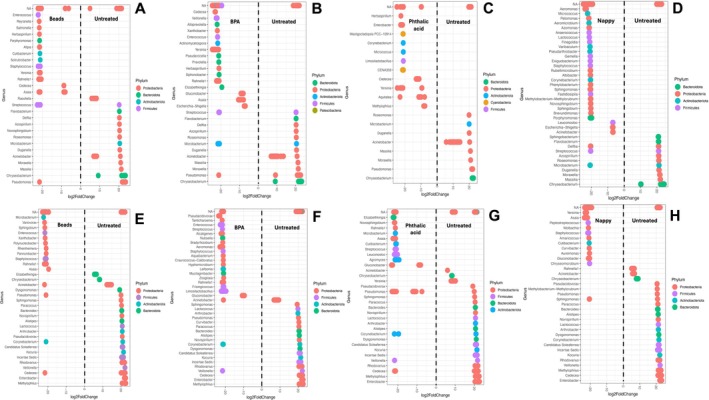
Differentially abundant bacterial genera in *Anopheles arabiensis* adults reared in plastic‐treated water. (A) Comparison of beads‐treated samples (left) versus untreated samples (right) in SENN. (B) Comparison of BPA‐treated samples (left) versus untreated samples (right) in SENN. (C) Comparison of phthalic acid‐treated samples (left) versus untreated samples (right) in SENN. (D) Comparison of nappy‐treated samples (left) versus untreated samples (right) in SENN. (E) Comparison of beads‐treated samples (left) versus untreated samples (right) in SENN‐DDT. (F) Comparison of BPA‐treated samples (left) versus untreated samples (right) in SENN‐DDT. (G) Comparison of phthalic acid‐treated samples (left) versus untreated samples (right) in SENN‐DDT. (H) Comparison of nappy‐treated samples (left) versus untreated samples (right) in SENN‐DDT. Numbers on either side of the 0 represent log2fold change of bacterial genera that are significantly different *α* = 0.01. Each dot represents a single OTU.

Differential abundance is a pairwise comparison of the difference in abundance of bacterial genera between two groups. The abundance of treated samples was compared to that of untreated samples, and those with a log2 fold change with an alpha value of under 0.01 were plotted. In general, the SENN strain had more differentially abundant genera in the plastic‐treated samples. When comparing the beads‐treated SENN to the untreated SENN, there were 13 more differentially abundant genera in the bead treatment compared to 12 in the untreated samples (Figure 5A). In the BPA‐treated SENN, there were 16 differentially abundant genera compared to 9 in the untreated SENN (Figure 5B). In the phthalic acid–treated SENN, there were 11 genera differentially abundant compared to 8 in the untreated SENN (Figure 5C). In nappy‐treated SENNs, there were 27 differentially abundant genera compared to 8 in the untreated SENN (Figure 5D).

This pattern was generally reversed in the SENN‐DDT strain. The beads‐treated SENN‐DDT had 11 differentially abundant genera compared to 18 in the untreated SENN‐DDT (Figure 5E). The BPA‐treated SENN‐DDT samples had 19 differentially abundant genera compared to 17 in untreated SENN‐DDT (Figure 5F). The phthalic acid–treated SENN‐DDT samples had 10 differentially abundant genera compared to 18 in the untreated SENN‐DDT samples (Figure 5G). For the nappy‐treated SENN‐DDT samples, 11 bacterial genera were differentially abundant compared to 22 in the untreated SENN‐DDT samples (Figure 5H).

## 4 Discussion

Although the challenge of plastic pollution is well documented (Welden 2020), the focus tends to be on marine environments. The effects on freshwater organisms, particularly insects, are less well described. Of the few descriptions of plastic on insects, the focus was on *Aedes* and *Culex* mosquitoes, and these studies focused on the effects of microplastics (Al‐Jaibachi et al. 2018, 2019; Edwards et al. 2023; Griffin et al. 2023; J. Li et al. 2024). This study focused on the major malaria vector, *An. arabiensis*, and on plastic derived from natural sources (latex beads), plastic additives (BPA, phthalic acid) and a degraded common plastic pollutant (disposable nappies). These plastic sources were chosen to represent a common pollutant in general, as well as the breeding sites of the *An. gambiae* complex in South Africa (Kordecki et al. 2022; Muringaniza et al. 2023; Schenck et al. 2023; Rosser et al. 2024). As such, this study is a generalised study on the effect of plastic pollution on the mosquito microbiome rather than a study of microplastics alone.

Furthermore, the effects of an insecticide‐resistant phenotype are considered, as this is known to affect the gut microbial composition (Dada et al. 2018; Singh et al. 2022). However, it was found that the insecticide‐selected phenotype (SENN‐DDT) had a limited effect on the alpha and beta diversity of microbial composition after treatment. In general, the different plastic treatments had a limited effect on the alpha diversity in both strains. In the SENN strain, the only differences in alpha diversity were between untreated and specific treatments rather than between the treatments themselves. In the SENN‐DDT strain, the nappy‐treated specimens had greater species richness than bead and BPA treatments, but not the untreated. In terms of species diversity in SENN‐DDT, BPA treatment was notable as having greater diversity than the control and bead treatment. Although there is not a large precedent for comparison, it is worth noting that microplastic treatment increased alpha diversity in both *Ae. albopictus* and *Ae. aegypti* (Edwards et al. 2023). The only notable change in richness was in the nappy treatment in SENN‐DDT, and this change may be analogous to the findings in *Aedes*. Additionally, a plausible explanation is that microplastics provide surfaces upon which microbial colonisation can occur. This creates what is known as the “plastisphere” (Zettler et al. 2013; Dussud et al. 2018). Therefore, upon microplastic ingestion, the plastisphere potentially plays a role in increasing the microbial communities present in the gut microbiota (Huang et al. 2022; Hu et al. 2022; Fackelmann et al. 2023). Therefore, it is possible that the nappy fragments served as a surface for bacterial growth, thus resulting in an increase in species richness within the midgut microbiota. In both the present study and the *Aedes* study, plastic treatment altered beta diversity. The effect of the different treatments had a significant effect on beta diversity, but this effect was not strain‐specific. This suggests that the insecticide‐resistant phenotype plays a limited role in affecting the changes in diversity seen after plastic treatment.

When examining the relative abundance of the five most common bacterial genera, the untreated samples were the most distinct in both strains. In terms of the treatments, for both strains, the beads and BPA‐treated mosquitoes were more similar to each other than to the nappy‐treated and phthalic acid–treated mosquitoes, which were similar to each other. The dominant genera in both strains were *Rahnella*, *Elizabethkingia* and *Acinetobacter*. It is notable that microplastic pollution increased the abundance of *Elizabethkingia* in both *Ae. albopictus* and *Ae. aegypti* (Edwards et al. 2023) as well as *Cx. quinquefasciatus* (J. Li et al. 2024). This is notable as *Elizabethkingia* has been known to form biofilms on plastic surfaces (Jacobs and Chenia 2011), suggesting that changes in this genus may be directly due to the interactions with plastic and the bacteria present on the plastic surfaces. When examining the overlapping species, it is notable that there were fewer species shared among treatments than the number of species unique to the treatments. This was also observed in *Ae. aegypti* and *Ae. albopictus* (Edwards et al. 2023). Furthermore, it is when looking at specific genera and species, rather than overall diversity, that the effect of insecticide‐resistant phenotype starts coming into effect. In the SENN strain, the nappy‐treated samples had the most unique bacterial species, followed by phthalic acid, BPA, untreated and beads. In contrast, for the SENN‐DDT strain, the untreated samples had the most unique species, followed by phthalic acid, BPA, nappy treatment and beads treatment.

When looking at differential abundance, there is a clear pattern in SENN where the treatment resulted in more abundant genera, while in SENN‐DDT the untreated samples had more differentially abundant genera when compared to the treatments. This was also a pattern that was observed when larvae were reared in metal‐treated water (Singh et al. 2024). This pattern may be related to the insecticide‐resistant phenotype. SENN‐DDT larvae typically survived better in more toxic pollutants than SENN (Oliver and Brooke 2018b, 2018a). This suggests that the elevated detoxification enzymes contributed to the improved survival in SENN‐DDT, but SENN did not have a protective effect. The increased abundance of bacteria, particularly pesticide and plastic‐degrading bacteria, in treated SENN suggests that this may be a response to the pollutant. This is particularly notable as this has been observed before in the same strains. Thus, the finding suggests that in the absence of an established metabolic detoxification system, bacteria could protect the larvae from toxicants, as this effect carries over to the adult.

Plastics are associated with enriched microbial growth (Wu et al. 2019; Sheridan et al. 2022), but bacteria are also capable of degrading plastics (Cai et al. 2023). It should therefore not be surprising that there was an increased abundance of bacterial genera and species associated with plastic degradation, which were dominant in the plastic‐treated samples. The most common plastic‐degrading genera found in both strains were *Acinetobacter* (Mohanan et al. 2020), *Corynebacterium* (Helleckes et al. 2023), *Cutibacterium* (Park et al. 2021), *Enterococcus* (Hou and Majumder 2021), *Gluconobacter* (Kim et al. 2019), *Herbapirillum* (X. Li et al. 2018), *Microbacterium* (Sun et al. 2022), *Pseudomonas* (Wilkes and Aristilde 2017), *Sphingomonas* (Pinyakong et al. 2004), *Staphylococcus* (Nowak et al. 2011), *Streptococcus* (Venkatesh et al. 2021) and *Veillonella* (Zhu et al. 2023). It is worth noting that nappy treatment in SENN resulted in the most plastic‐degrading bacteria, while BPA resulted in the most pesticide‐degrading bacteria.

However, larval exposure to a pollutant not only results in an enhanced response to the specific xenobiotic but xenobiotics in general (Oliver and Brooke 2018b, 2018a). This holds true in the microbial response as well. Larval exposure to metal increased insecticide resistance in the strains, even after a new generation bred in unpolluted water. This was due to a generalised increase in metabolic detoxification enzymes (Jeanrenaud et al. 2020). However, there was also an increase in pesticide‐degrading bacterial genera in the strains bred in metal‐polluted water. This increase in pesticide‐degrading bacteria was also found in F1 *An. arabiensis*, demonstrating that this increased effect was present in wild mosquitoes as well (Singh et al. 2024). The most common genera associated with pesticide‐degrading bacteria found in the treatments were *Acinetobacter* (Wang et al. 2021), *Cutibacterium* (Muturi et al. 2021), *Corynebacterium* (Muturi et al. 2021), *Herbaspirillum* (Venkatachalam et al. 2023), *Microbacterium* (de Almeida et al. 2017), *Micrococcus* (Dar and Kaushik 2022), *Pseudomonas* (Dada et al. 2019; Wang et al. 2021; Gómez‐Govea et al. 2022) and *Sphingomonas* (Lv et al. 2023). Like with the plastic‐degrading bacteria, in SENN, the nappy treatments resulted in the most bacterial genera, and BPA treatment resulted in the most pesticide‐degrading genera.

An additional concern is the potential effect of plastic pollution on vector competence. This capacity would be modulated by alteration of the immune system, which can occur by two mechanisms. The first mechanism is the alteration of the balance of bacteria associated with protection against *Plasmodium*. This balance was not widely affected by any of the experimental treatments. When looking at differential abundance, there were not many genera found abundant in either the treated or untreated groups. In the SENN strain, although few, most *Plasmodium* protective genera were differentially abundant in the untreated strains. When analysing the beads treatment, there were two *Plasmodium* protective genera in the untreated (*Delftia* and *Acinetobacter*), one shared (*Pseudomonas*) and two present in the bead treatment (*Asaia* and *Cedecea*). In phthalic acid, there were two in the untreated (*Acinetobacter* and *Pseudomonas*) and two in the phthalic acid treatment (*Enterobacter* and *Cedecea*). The nappy treatment had two (*Aeromonas* and *Acinetobacter*) and one shared (*Delftia*) and one in the untreated (*Sphingobacterium*). When comparing BPA, there were two *Plasmodium* protective genera in the untreated (*Acinetobacter* and *Delftia*), one shared (*Pseudomonas*) and three in the BPA treatment (*Elizabethkingia, Asaia* and *Cedecea*).

This was similar in SENN‐DDT. There were two *Plasmodium* protective genera differentially abundant in the untreated (*Enterobacter* and *Elizabethkingia*) compared to the one in beads (*Asaia*), and three genera were shared (*Pseudomonas*, *Acinetobacter* and *Cedecea*). Three *Plasmodium* protective genera were differentially abundant in the untreated (*Enterobacter*, *Cedecea* and *Pseudomonas*), one was shared (*Acinetobacter*), and two were differentially abundant in BPA treatment (*Bradyrhizobium* and *Aeromonas*). When comparing phthalic acid, two genera were differentially abundant in the untreated (*Acinetobacter* and *Enterobacter*), two were shared (*Pseudomonas* and *Cedecea*) and two were differentially abundant in phthalic acid (*Elizabethkingia* and *Asaia*). Four genera were differentially abundant in the untreated (*Acinetobacter*, *Cedecea*, *Enterobacter* and *Pseudomonas*) and two were differentially abundant in the nappy treatment (*Asaia* and *Wolbachia*). Although this is not a clear pattern, there appear to be fewer protective genera in the treated mosquitoes than in the untreated mosquitoes. This observation needs to be examined further, particularly in wild specimens. Therefore, it is still unclear as to whether microbial manipulation by plastic exposure will directly alter mosquito vectorial capacity (Jones et al. 2024).

What is known is that microplastic exposure does alter the mosquito's immune response. Microplastic ingestion has been demonstrated to increase phenoloxidase activity as well as oxidative stress (Silva, Beleza, et al. 2021; Silva, Patrício Silva, et al. 2021). Both of these factors would reduce *Plasmodium* infection. Inflammatory‐type responses occur in insects as this is an innate response (Chain and Anderson 1983). Several bacteria associated with inflammatory responses occur after plastic exposure. The most common of these genera found in the treatments are *Acinetobacter*, *Aeromonas*, *Corynebacterium*, *Cutibacterium*, *Enterococcus*, *Gluconobacter*, *Limnosilactobacilus*, *Pseudomonas*, *Sphingomonas*, *Staphylococcus*, *Streptococcus* and *Veillonella*. If inflammatory responses are more common in plastic‐treated mosquitoes, this could potentially reduce the capacity to transmit parasites.

There were two notable findings in this study. The genus *Afpia* was found differentially abundant in beads‐treated SENN. The first report of *Afpia* was in *Aedes* in 2022 (da Silva et al. 2022). As such, to the best of our knowledge, this is the first report of *Afpia* in the *Anopheles* mosquitoes. The second finding is the differential abundance of *Wolbachia* and *Asaia* in nappy‐treated SENN‐DDT. This is notable in two regards. The first is that *Wolbachia* was found in a laboratory strain of *An. arabiensis*, and this genus is not common in *Anopheles* mosquitoes, particularly in laboratory strains where the microbiota is reduced (Chrostek and Gerth 2019; Wong et al. 2020). *Asaia* has been known to impede the transmission of *Wolbachia*, so finding both of these differentially abundant in treated SENN‐DDT is unusual.

It must be noted that this study has several limitations. We do not know whether the concentrations of plastic pollutants used in this study are relevant to mosquito breeding sites, particularly within Southern Africa. This is a general problem with plastic studies, as although there are records of microplastic and additive pollutants in water, the concentrations of these pollutants in the temporary breeding sites are unclear (Jones et al. 2024). Recently, a study characterising the presence of MPs within mosquito oviposition sites was conducted and suggested a more plausible approximate concentration of 10–25 MPs/mL (McConnel et al. 2024). Nevertheless, there remains a significant knowledge gap regarding the concentration of plastic additives present within mosquito breeding sites. Furthermore, the applicability of these findings within a South African context remains uncertain and requires further investigation. Therefore, the concentrations used allowed for the survival of the strains to allow reasonable numbers of mosquitoes. As such, they may not be fully ecologically relevant. Additionally, a comprehensive compositional analysis of the artificially degraded nappies and the MP beads used in this study is imperative to further clarify the effect of these plastics on the mosquito microbiota. Crucially, this study must be replicated with wild *An. arabiensis*. It has been demonstrated that wild mosquitoes have a greater bacterial diversity than their laboratory‐reared counterparts (Saab et al. 2020). Therefore, despite the large‐scale changes observed, the findings are likely to be an underrepresentation of the changes in wild microbiota. Finally, further studies are crucial to determine the exact effects of plastic on the immune system of *An. arabiensis*.

In conclusion, larval exposure to plastic pollution had a large‐scale effect on the microbiota of adult *An. arabiensis*. Although the insecticide‐selected phenotype was not as important as treatment in altering diversity in general, it did affect the patterns of bacterial abundance in the adults. The insecticide‐unselected strain had a greater abundance of bacteria in response to degraded plastic, while the insecticide‐selected strain had more genera abundant in the untreated group. This suggests that the increased abundance of bacteria in response to plastic treatment may serve a protective function in the absence of a pre‐existing resistant phenotype. Larval exposure to plastic products increased the abundance of genera associated with both plastic and insecticide degradation. The abundance of *Plasmodium*‐protective genera was not significantly affected by the exposure to plastics, but bacteria associated with inflammation were significantly more abundant. These results suggest that larval exposure to plastic products has the capacity to alter microbial composition in a manner that could have epidemiological consequences.

## Author Contributions

**Shristi Misser:** investigation, writing – review and editing, software, formal analysis, data curation, visualization. **Chia‐Yu Chen:** supervision, formal analysis, software, writing – review and editing. **Arshad Ismail:** resources. **Shüné V. Oliver:** supervision, conceptualization, funding acquisition, writing – original draft, project administration, resources.

## Ethics Statement

The animal study protocol was provided with an ethics waiver by the Animal Research Ethics Committee of the University of the Witwatersrand as all work was conducted on Dipteran invertebrates (protocol code: S Oliver 29‐02‐2024‐O approved on 29 February 2024).

## Conflicts of Interest

The authors declare no conflicts of interest.

## Supporting information


**Figure S1:** Rarefaction analysis illustrating sequencing depth and species richness within individual samples. The corresponding rarefaction curve depicts the relationship between sample size and observed species richness, specifically the amplicon sequence variants (ASVs) derived from the 16S rRNA gene amplicons across the different treatments. The *x*‐axis represents the number of sequences sampled (sample size), while the *y*‐axis indicates the cumulative number of observed species (ASVs). Each curve corresponds to an individual sample. The attainment of a plateau by the curves suggests that the sampling depth was sufficient to encompass the majority of microbial diversity within each sample.

## Data Availability

The data presented in this study are available on request from the corresponding author.
