# Supplementary figures and images for: The Effect of Larval Exposure to Plastic Pollution on the Gut Microbiota of the Major Malaria Vector Anopheles arabiensis Patton (Diptera: Culicidae)
Source: Environ Microbiol Rep. 2025 Aug 3;17(4):e70169. doi: 10.1111/1758-2229.70169 (PMC12318828; doi:10.1111/1758-2229.70169)

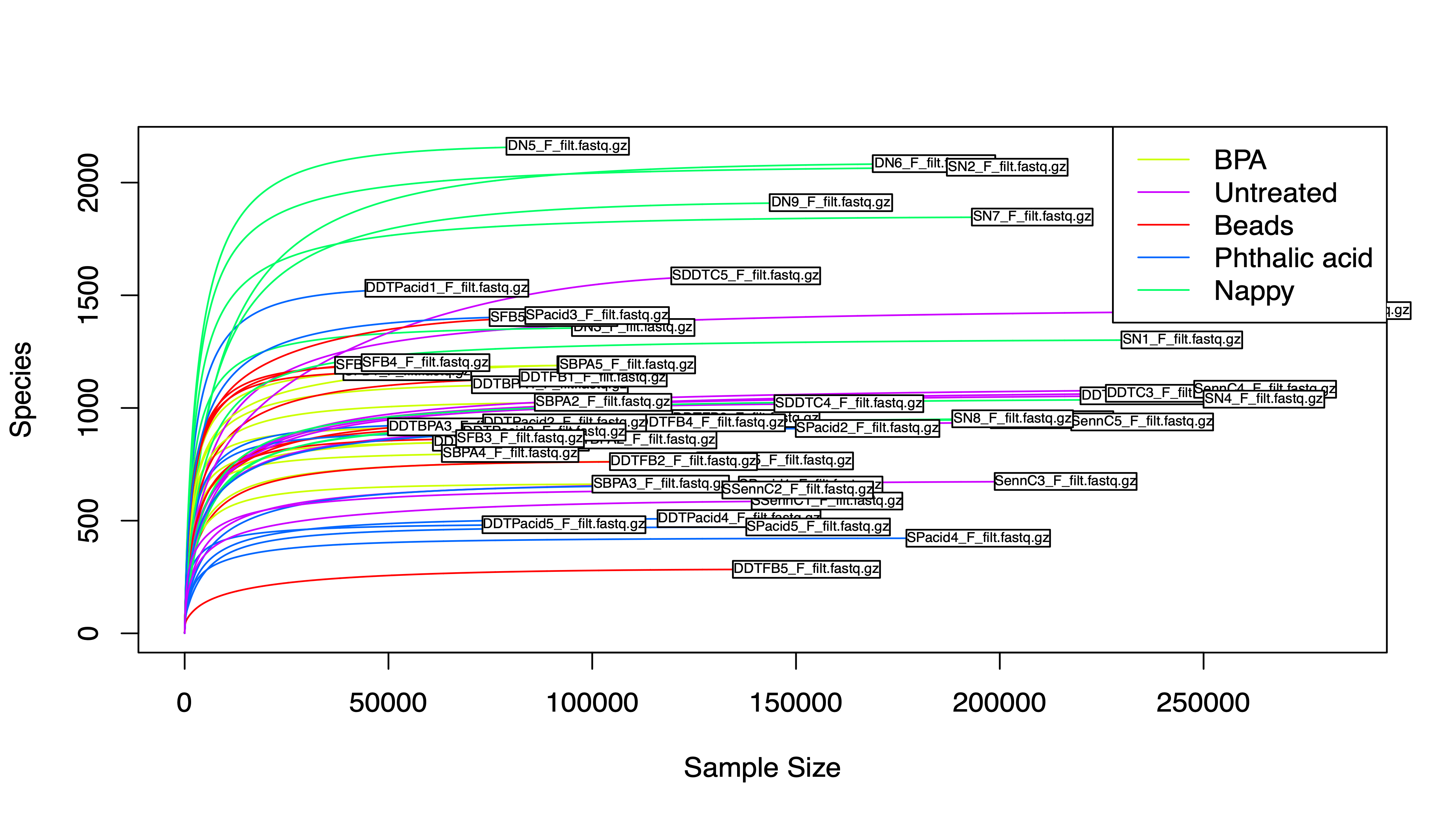

Supplement: Supplementary file 1 — Figure S1: Rarefaction analysis illustrating sequencing depth and species richness within individual samples. The corresponding rarefaction curve depicts the relationship between sample size and observed species richness, specifically the amplicon sequence variants (ASVs) derived from the 16S rRNA gene amplicons across the different treatments. The x‐axis represents the number of sequences sampled (sample size), while the y‐axis indicates the cumulative number of observed species (ASVs). Each curve corresponds to an individual sample. The attainment of a plateau by the curves suggests that the sampling depth was sufficient to encompass the majority of microbial diversity within each sample. [file EMI4-17-e70169-s001.tiff]
